# Mentorship in field epidemiology training programs in the eastern mediterranean region: mentors' characteristics, perceived challenges, and training needs

**DOI:** 10.3389/fpubh.2025.1669305

**Published:** 2025-12-10

**Authors:** Yousef Khader, Mohannad Al Nsour, Abdulla Bin-Ghouth, Randa Mohammed S. Nooh, Ruba Kamal Alsouri, Ayman Bani Mousa, Hajer Letaief, Yasir Younis Majeed, Haitham Bashier, Khwaja Mir Islam Saeed, Hassan Chrifi, Muntasir Mohammed Osman, Tahmina Shirin, Mumtaz Ali Khan, Mohammed Akrim

**Affiliations:** 1 Department of Public Health, Jordan University of Science and Technology, Irbid, Jordan; 2 The Eastern Mediterranean Public Health Network, Amman, Jordan; 3 Yemen National Public Health Institute, Aden, Yemen; 4 Saudi Field Epidemiology Training Program, Department of Population Health, Ministry of Health, Riyadh, Saudi Arabia; 5 Epidemic Administration Directorate, Jordan Ministry of Health, Amman, Jordan; 6 National Observatory of New and Emerging Diseases, Faculty of Medicine of Tunis, Tunis, Tunisia; 7 Field Epidemiology Training Program, Ministry of Health, Baghdad, Iraq; 8 Afghanistan National Public Health Institute, Ministry of Public Health, Kabul, Afghanistan; 9 National School of Public Health, Ministry of Health and Social Protection, Rabat, Morocco; 10 Sudan Federal Ministry of Health, Khartoum, Sudan; 11 Institute of Epidemiology, Disease Control & Research, Dhaka, Bangladesh; 12 Pakistan Field Epidemiology Training Program, Islamabad, Pakistan; 13 National School of Public Health, Rabat, Morocco

**Keywords:** field epidemiology, mentorship, EMPHNET, challenges, training needs

## Abstract

**Introduction:**

Despite its significance, mentorship within Field Epidemiology Training Programs (FETP) remains underexplored. This study aimed to assess the demographic and professional profiles of FETP mentors, their mentorship practices, challenges, and training needs within the Eastern Mediterranean Region (EMR).

**Methods:**

A cross-sectional survey was conducted among FETP mentors across nine EMR countries. A structured questionnaire, developed with expert input and pre-tested for clarity, covered demographics, challenges, training needs, satisfaction, and perceived impact. Data were collected via an online survey platform.

**Results:**

Of 148 mentors approached, 129 responded (87.2%). Most were male (75.2%) and aged 40–50 years (41.1%). Over half (51.1%) had more than 6 years of professional experience, while 38.0% had only 0–2 years of mentoring experience. Epidemiology (50.4%) and public health (31.0%) were the most common fields. Most (74.4%) had formal mentorship training, though only 47.3% did so recently. Time management was a common challenge, with 68.0% of mentors reporting difficulty managing time for mentorship activities either often or sometimes. Nearly half (49.2%) found available resources adequate to support their mentorship role. The majority (93.0%) expressed a need for more training, especially in mentoring techniques, communication, and leadership. Workshops were identified by 94.6% of respondents as the preferred method for mentor training. Overall, 59.7% rated their mentorship experience as “Good” and 27.1% as “Excellent.”

**Conclusion:**

The study findings underscore the need for structured mentor development, clear guidelines, dedicated time, and enhanced support to strengthen FETP mentorship and public health capacity.

## Introduction

The Field Epidemiology Training Program (FETP) is an in-service, on-the-job mentored training program designed to build the capacity of public health professionals in disease outbreak investigation, surveillance system strengthening, and applied epidemiological research (1–3). Unlike traditional academic programs, FETP emphasizes experiential learning, enabling trainees to work directly within health departments, applying epidemiological methods in real-world settings under the guidance of experienced mentors. Following the learning-by-doing approach (4, 5), FETPs allocate 75% of training time to field placements, ensuring that participants gain hands-on experience under mentor supervision (6). FETP graduates play a pivotal role in global public health efforts, contributing significantly to disease control (7–13).

Mentorship is a cornerstone of professional development in FETPs. Effective mentorship not only enhances technical competencies but also fosters critical thinking, leadership, and problem-solving skills essential for responding to public health threats. Strong mentorship expands professional networks, creates career development opportunities, and boosts trainees' confidence and resilience (14, 15). By providing guidance, support, and knowledge transfer in real-world settings, mentorship plays a crucial role in shaping future epidemiologists.

Research has consistently shown that high-quality mentorship improves career satisfaction, fosters professional growth, and enhances workforce retention (15, 16). Given FETPs' strong emphasis on the learning-by-doing approach, mentors are central to the success of these programs. Mentors play a vital role in developing trainees' analytical and investigative skills, ensuring they become proficient in data collection, analysis, and interpretation to support evidence-based public health decision-making (14–17). They oversee trainees' progress, support them in conducting field investigations, and guide them in preparing the necessary outputs to complete program requirements (17). Mentors ensure that their mentees develop core competencies to detect, prevent, and contain infectious disease threats, safeguarding both human and animal health. Additionally, mentors serve as career advisors, role models, and facilitators of professional networking opportunities. Their willingness to involve mentees in outbreak investigations or other fieldwork experiences is often crucial to trainees' professional development.

Despite the recognized importance of mentorship in public health training, research on the specific competencies required for FETP mentors remains limited (15). Most existing studies on mentorship focus on medical residency programs or academic institutions, with fewer addressing mentorship in field-based applied epidemiology programs. Despite its significance, mentorship within FETPs—particularly in the Eastern Mediterranean Region (EMR)—remains underexplored. There is limited understanding of the characteristics, experiences, and challenges faced by FETP mentors in the region. To address these gaps, this study assessed the demographic and professional profiles of FETP mentors, their mentorship practices, challenges, and training needs within the EMR. The findings are expected to inform the design of targeted mentorship training programs, improve institutional support mechanisms, and enhance the overall effectiveness of mentorship in FETPs. This study will contribute to the growing body of literature on public health mentorship, offering insights relevant to workforce development in applied epidemiology programs worldwide.

## Methods

### Study design

This study employed a cross-sectional survey design to assess the experiences, challenges, training needs, and satisfaction levels of mentors in Field Epidemiology Training Programs (FETPs) across 9 countries in the EMR. The study specifically targeted public health professionals who serve as mentors, providing guidance and supervision to FETP trainees. To participate, mentors had to meet the following criteria: they must have been actively engaged as mentors within one of the 9 participating countries, have provided supervision, guidance, or training to FETP trainees within the past 2 years, and voluntarily agreed to complete the survey after being informed about the study's purpose and confidentiality measures. All eligible mentors from the participating FETPs were invited to complete the survey. To encourage broad participation and maximize response rates, program directors from the 9 countries were contacted and asked to distribute the survey link to mentors within their programs, ensuring official program endorsement and facilitating higher engagement. Additionally, the study team sent direct email invitations to mentors using an existing database, which helped reach those who may not have been contacted via their program directors.

### Questionnaire

The survey questionnaire was designed to comprehensively assess the experiences, challenges, and training needs of mentors within FETPs. Its development followed a structured process. A thorough review of existing mentorship evaluation frameworks, common challenges in epidemiology training programs, and best practices in mentor development informed the identification of key domains relevant to mentorship effectiveness. A panel of experts, including senior epidemiologists, program coordinators, and professionals with extensive FETP mentorship experience, provided input on the questionnaire's content and structure. Items that were unclear, redundant, or ambiguous were revised or removed. The revised version was pre-tested with a small sample of mentors (*n* = 10) to evaluate clarity, ease of understanding, and response time. Based on feedback, minor modifications were made to improve wording and format.

The questionnaire consisted of five sections. The first section gathered demographic and professional background information, including age, gender, country of residence, job title, organization, years of experience in epidemiology and mentorship, educational background, areas of expertise, and prior mentorship training. The second section examined common barriers to effective mentorship, such as time constraints, resource availability, organizational support, communication issues, unclear guidelines, mentee engagement, and feedback mechanisms. Mentors rated the frequency and impact of these challenges on a five-point Likert scale. The third section identified areas where mentors required additional training and professional development, including preferred training topics (e.g., communication skills, conflict resolution, mentoring techniques, cultural competency) and delivery methods (e.g., workshops, online courses, peer learning, one-on-one coaching). Barriers to training participation, such as time limitations and lack of resources, were also assessed. The fourth section asked mentors to rate their overall satisfaction with their mentoring experience, organizational support, communication with mentees, availability of training and development opportunities, and recognition or incentives. It also explored mentors' perceptions of their impact on their mentees' professional growth.

### Data collection

The survey was administered electronically through a secure online platform, providing participants with a link to complete the questionnaire at their convenience. The questionnaire was available in English, with clear instructions provided to ensure accurate responses. The survey remained open for a predetermined period, and reminder emails were sent to encourage participation.

### Ethical considerations

Ethical approval for the study was obtained from the Institutional Review Board at EMPHNET. Ethical principles were upheld throughout the study to ensure the protection of participants' rights. Informed consent was obtained from all respondents, with explicit communication regarding the survey's purpose, the voluntary nature of participation, and the privacy protections in place. Participants were informed that they could skip any questions or exit the survey at any time without any consequence. All responses were kept anonymous, and data confidentiality was rigorously maintained throughout the study.

### Data analysis

Frequencies and proportions were calculated for categorical variables. Data were analyzed using IBM SPSS, version 24.

## Results

### Participants' characteristics

A total of 129 mentors (87.2%) completed the questionnaire. The majority of FETP mentors were male (75.2%), with most falling within the 40–50 age group (41.1%), followed by those under 40 (35.7%) (Table 1). Nationally, the largest proportions are from Afghanistan (25.6%), Iraq (20.2%), and Yemen (12.4%). In terms of experience, 51.1% had more than 6 years of professional experience, 38.0% had 0–2 years of mentoring experience, and 32.6% had 3–5 years. Of all mentors, 46.5% held a master's degree, 22.5% held a Doctorate, and 20.2% a bachelor's degree. Mentors represented all FETP tiers (Frontline, Intermediate, and Advanced). Epidemiology (50.4%) and Public Health (31.0%) were the most common fields of study among the mentors.

**Table 1:** Sociodemographic characteristics and qualifications of FETP mentors.

**Variable**	**Frequency**	**Percent**
**Gender**		
Female	32	24.8
Male	97	75.2
**Age (year)**		
< 40	46	35.7
40–50	53	41.1
>50	30	23.3
**Nationality**		
Afghanistan	33	25.6
Bangladesh	20	15.5
Iraq	26	20.2
Jordan	5	3.9
Morocco	7	5.4
Pakistan	9	7.0
Sudan	8	6.2
Tunisia	5	3.9
Yemen	16	12.4
**Years of experience (years)**		
0–2	13	10.1
3–5	33	25.6
6–10	35	27.1
11–15	17	13.2
>15	31	24.0
**Years of experience as a mentor (year)**		
0–2	49	38.0
3–5	42	32.6
6–10	28	21.7
11–15	6	4.7
>15	4	3.1
**Highest degree obtained**		
Bachelor's degree	26	20.2
High diploma	7	5.4
Doctorate	29	22.5
Master's degree	60	46.5
Other	7	5.4
**Field of study**		
Epidemiology	65	50.4
Public Health	40	31.0
Medicine	11	8.5
Veterinary Science	4	3.1
Environmental Health	2	1.6
Others	7	5.4

### Mentors' professional development, areas of expertise, and experience

The majority of mentors (74.4%) had received formal mentorship training, though only 47.3% had done so in the last 2 years (Table 2). Participation in continuous professional development (CPD) related to mentorship in the past 2 years was reported by 72.1% of mentors. About half of the mentors (54.3%) had mentored 1–5 mentees, while 46% had mentored more than five. Field epidemiology (87.6%) was the predominant area of expertise, followed by infectious diseases (72.1%) and public health surveillance (66.7%). Motivations for mentoring included professional development (74.4%), passion for teaching (73.6%), and a desire to give back to the community (64.3%). The primary goal as mentors was to enhance mentees' technical skills (95.3%), along with supporting personal development (76.7%) and providing career guidance (59.7%).

**Table 2:** Mentors' professional development, areas of expertise, and experience.

**Variable**	***n***	**%**
Received any formal training in mentorship	96	74.4
Received any formal training in mentorship in the last two years	61	47.3
Have participated in any continuous professional development (CPD) activities related to mentorship in the last two years	93	72.1
**The number of mentees a mentor has guided**		
1–5	70	54.3
6–10	22	17.1
11–20	19	14.8
>20	18	14.0
**Types of mentorships provided**		
One-on-one mentorship	90	69.8
Group mentorship	67	51.9
Online mentorship	58	45.0
In-person mentorship	65	50.4
**Areas of expertise in epidemiology**		
Field epidemiology	113	87.6
Infectious Diseases	93	72.1
Public health surveillance	86	66.7
One health	56	43.4
Biostatistics	51	39.5
Non-communicable diseases	39	30.2
Environmental health	32	24.8
Health policy and management	27	20.9
Water, sanitation, and hygiene (WASH)	22	17.1
Digital health, mHealth, eHealth	11	8.5
Motivators to become a mentor		
Professional development	96	74.4
Passion for teaching and mentoring	95	73.6
Desire to give back to the community	83	64.3
Organizational requirement	51	39.5
**The primary goals of a mentor**		
Enhancing mentees' technical skills	123	95.3
Supporting personal development	99	76.7
Providing career guidance	77	59.7
Fostering professional networks	64	49.6

### FETP mentors' self-reported challenges

The findings revealed several challenges and needs that mentors reported encountering in their roles (Table 3). A majority of FETP mentors (68.0%) often or sometimes face difficulties managing their time for mentorship activities, with only 6.3% indicating frequent issues. The leading cause of these time management challenges, as reported by 78.3% of respondents, is a high workload, followed by conflicting responsibilities for 33.3%.

**Table 3:** Challenges faced by FETP mentors.

**Variable**	***n***	**%**
**Difficulties in managing time for mentorship activities**		
Often	8	6.3
Sometimes	79	61.7
Rarely	41	32.0
**Resource adequacy to support the mentorship role**		
Adequate	63	49.2
Neutral	45	35.2
Inadequate	20	15.6
**Organizational support for mentorship activities**		
Good	71	55.5
Fair	37	28.9
Poor	20	15.6
**Communication challenges with mentees**		
Often	13	10.2
Sometimes	52	40.6
Rarely	63	49.2
**Clarity of the guidelines**		
Clear	89	69.5
Neutral	29	22.7
Unclear	10	7.8
**Mentee engagement and motivation**		
Engaged and motivated	101	78.9
Neutral	25	19.5
Disengaged and unmotivated	2	1.6
**Receiving constructive feedback from mentees**		
Regularly	41	32.3
Occasionally	60	47.2
Rarely	26	20.5
**Receiving constructive feedback from the organization**		
Regularly	35	27.8
Occasionally	44	34.9
Rarely	47	37.3

Regarding the resources available to support their mentorship roles, 49.2% of mentors reported them as adequate, while 15.6% found them inadequate. The most reported deficiencies include a lack of financial support (57.4%), technological tools (49.6%), access to research data (38.8%), and educational materials (32.6%).

Organizational support for mentorship was considered good by 55.5% of mentors, though 15.6% rated it as poor. Mentors identified several forms of organizational support as crucial for effective mentorship, with the most significant need being training and development opportunities, as highlighted by 77.5% of respondents. Other needs include administrative assistance (51.2%), clear policies and guidelines (49.6%), and recognition and incentives (48.8%).

Communication with mentees was reported to be challenging for some mentors, with 10.2% stating that it occurs often and 40.6% reporting that it happens sometimes. The primary issues include mentees' lack of responsiveness (54.3%) and technological barriers (43.4%), while smaller proportions of mentors face language barriers (7.8%) and cultural differences (7.0%).

While most mentors (69.5%) found the guidelines and expectations for mentorship clear, 57.4% indicated that roles and responsibilities were unclear. Mentee engagement was high, with 78.9% reporting engaged and motivated mentees. However, external commitments (46.5%), personal issues (42.6%), and a lack of understanding of the benefits of mentorship (31.0%) were cited as factors impacting mentee motivation. Additionally, 28.7% of mentors reported that mentees lacked interest in the program. The mentorship process itself was seen as requiring better definition by 55.0% of respondents, while 45.7% pointed to unclear evaluation criteria and 26.4% highlighted concerns regarding the clarity of goals and objectives.

In terms of feedback, 32.3% of mentors regularly received constructive feedback from their mentees, while 27.8% received feedback from the mentees' organizations. However, 62.0% of mentors reported the absence of formal feedback systems as a significant issue. Additionally, 28.7% mentioned inconsistent feedback, 25.6% faced difficulties in providing honest feedback, and 14.7% encountered challenges in receiving constructive criticism from their mentees.

### Training needs

The findings from the training needs assessment among FETP mentors reveal a strong demand for further development, with 93.0% of respondents indicating a need for additional training as mentors (Table 4). Among specific areas of training, mentoring techniques and best practices were the most sought after (62.0%), followed by effective communication skills (61.2%) and leadership skills (60.5%). Feedback and evaluation techniques were also important for 55.8% of respondents, while time management and emotional intelligence were cited by 46.5% and 42.6%, respectively. Conflict resolution was noted by 39.5%, and cultural competency by 18.6%.

**Table 4:** Training needs assessment among FETP mentors.

**Variable**	***n***	**%**
Need further training or development as a mentor	120	93.0
**Specific areas are most beneficial for the role of a mentor**		
Mentoring techniques and best practices	80	62.0
Effective communication skills	79	61.2
Leadership skills	78	60.5
Feedback and evaluation techniques	72	55.8
Time management	60	46.5
Emotional intelligence	55	42.6
Conflict resolution	51	39.5
Cultural competency	24	18.6
**Preferred training methods**		
Workshops	122	94.6
Seminars	43	33.3
Peer learning groups	35	27.1
Online courses	34	26.4
One-on-one coaching	40	31.0
Self-paced learning modules	27	20.9
Webinars	17	13.2
**Desired training frequency**		
Quarterly	44	34.6
As needed	25	19.7
Annually	24	18.9
Biannually	23	18.1
Monthly	11	8.7
**Barriers to participating in training programs**		
Cost of training programs	85	65.9
Lack of time	54	41.9
Limited access to training resources	47	36.4
Lack of awareness of available training	50	38.8
Lack of organizational support	48	37.2
Geographical location	29	22.5
Inconvenient scheduling	27	20.9
**Rating the content and quality of the training programs that**		
**have been attended in the past**		
Excellent	38	30.9
Good	62	50.4
Fair	21	17.1
Poor	2	1.6
Suggested improvements for future training programs		
More practical examples and case studies	104	80.6
Interactive sessions	74	57.4
Follow-up support and coaching	74	57.4
Experienced trainers	69	53.5
Comprehensive materials and resources	62	48.1
Relevance of the training programs to the mentorship role		
Highly relevant	37	29.6
Relevant	64	51.2
Neutral	23	18.4
Highly irrelevant	1	0.8
**Preferred topics to be covered in future training programs**		
Advanced epidemiological methods	108	83.7
Data analysis and interpretation	94	72.9
Effective mentorship strategies	93	72.1
Public health leadership	82	63.6
Ethics in mentoring	52	40.3

Preferred training methods showed a significant inclination toward workshops, favored by 94.6% of respondents. Other methods, such as one-on-one coaching (31.0%), seminars (33.3%), and peer learning groups (27.1%), were less popular. Online courses and webinars were chosen by 26.4% and 13.2%, respectively, while self-paced learning modules appealed to 20.9%.

When it comes to the desired training frequency, quarterly sessions were the most preferred (34.6%), followed by training offered as needed (19.7%) and annually (18.9%). Lack of time was a significant barrier to participating in training programs for 41.9%, along with limited access to training resources (36.4%) and lack of organizational support (37.2%). Other barriers included lack of awareness about training opportunities (38.8%), inconvenient scheduling (20.9%), and geographical constraints (22.5%).

Regarding the content and quality of past training programs, 30.9% rated them as excellent, 50.4% as good, 17.1% as fair, and 1.6% rated the training as poor. Suggested improvements for future programs included incorporating more practical examples and case studies (80.6%), interactive sessions (57.4%), and comprehensive materials and resources (48.1%). Respondents also emphasized the importance of follow-up support and coaching (57.4%) and engaging experienced trainers (53.5%).

The relevance of training programs to the mentorship role was highlighted, with 51.2% of respondents considering them relevant and 29.6% rating them as highly relevant. Only 0.8% found the programs highly irrelevant.

For future training topics, advanced epidemiological methods were the top priority, indicated by 83.7% of respondents. Effective mentorship strategies (72.1%), data analysis and interpretation (72.9%), and public health leadership (63.6%) were also highly valued. Ethics in mentoring was reported by 40.3%, reflecting the diversity of training needs.

### Self-reported confidence in essential skills

The highest confidence was reported in mentoring techniques and best practices, with 74.4% of mentors expressing confidence in this area (Table 5). Other skills with high self-reported confidence include emotional intelligence (72.8%), time management (72.9%), providing feedback (72.1%), and cultural competency (72.1%). Mentors also reported a strong level of confidence in technical skills in epidemiology (71.4%) and leadership skills (69.7%). Communication skills were the area with the lowest confidence, though still a majority (68.2%) of mentors reported feeling confident in their communication abilities.

**Table 5:** Proportions of mentors who have self-reported confidence in key mentorship skills.

**Variable**	***n***	**%**
Mentoring techniques and best practices	96	74.4
Time management	94	72.9
Emotional intelligence	94	72.8
Providing feedback	93	72.1
Cultural competency	93	72.1
Technical skills in epidemiology	92	71.4
Leadership skills	90	69.7
Communication skills	88	68.2

### Mentors' satisfaction

Mentors were most satisfied with the communication and interaction with their mentees, with 82.9% reporting being satisfied or very satisfied (Table 6). Satisfaction with the impact on mentees' professional development was also high, at 75.9%. In terms of mentee progress and success, 77.5% of mentors were satisfied or very satisfied. However, satisfaction with the support received from the organization for the mentorship role was lower, with 57.4% expressing satisfaction.

**Table 6:** Proportion of mentors who reported being satisfied or very satisfied with mentorship aspects.

**Variable**	***n***	**%**
Communication and interaction with mentees	107	82.9
The progress and success of mentees	100	77.5
The impact on mentees' professional development	98	75.9
The support received from the organization for the mentorship role	74	57.4
The recognition and incentives provided for mentors	72	55.8
The training and development opportunities provided to the mentor	70	54.3
The feedback mechanisms in place for the mentorship role	68	52.8

Other areas, such as training and development opportunities provided to the mentor (54.3%), recognition and incentives for mentors (55.8%), and feedback mechanisms in place for the mentorship role (52.8%), had relatively lower satisfaction levels. In terms of recognition or incentives, 79.1% of mentors found professional development opportunities the most motivating, followed by certificates or awards (60.5%) and financial rewards (51.2%).

When rating their overall experience as a mentor in the Field Epidemiology Training program, 59.7% rated it as “Good”, 27.1% as “Excellent”, 10.9% as “Fair”, and only 0.8% as “Very Poor”.

## Discussion

This study highlights both the strengths of FETP mentorship and the challenges faced by mentors across nine countries in the EMR, offering insights into an essential yet underexplored component of applied epidemiology training. The findings demonstrate that mentors are experienced public health professionals with strong technical expertise. However, they often operate within systems that lack the institutional, financial, and structural support needed to maximize the effectiveness of mentorship. Most mentors expressed a clear need for additional training, particularly in mentoring techniques, communication, leadership, and feedback provision, to enhance their capacity to guide and support trainees. Despite these challenges, mentors reported high satisfaction with their mentorship experience and demonstrated strong intrinsic motivation, reflecting their commitment to sustaining public health capacity development in the region.

The mentor profile revealed in this study, experienced epidemiologists and public health practitioners, underscores the value of engaging mentors from within national public health systems. Their expertise in field epidemiology, infectious diseases, and surveillance aligns closely with the FETP learning model, which emphasizes the practical, field-based application of epidemiologic methods (1–4). This internal capacity is a major strength: mentors serve not only as technical experts but also as professional role models who transmit institutional knowledge, reinforce national surveillance priorities, and foster applied problem-solving skills among trainees.

Mentors' reported motivations, including professional development, passion for teaching, and service to their communities, mirror findings from other studies indicating that mentoring enhances mentors' professional identity, confidence, and job satisfaction (18, 19). FETP programs should capitalize on this intrinsic motivation by establishing mechanisms that recognize and strengthen mentors' roles. This could include opportunities to present their contributions at regional forums, participate in mentor networks that facilitate peer learning, and engage in professional development activities that promote ongoing growth and institutional recognition.

Despite these positive attributes, mentors face several structural and operational barriers that limit their effectiveness. The most prominent challenge reported was the lack of time for mentorship due to competing professional duties and heavy workloads. Similar challenges have been described in other field-based training contexts (20). Limited dedicated time leads to inconsistent mentor–mentee interactions and constrains the quality of supervision and feedback. Institutional solutions such as allocating protected mentorship time in job descriptions, setting clear performance expectations, and providing workload relief during mentorship cycles could substantially improve engagement and accountability. Ministries of Health and FETP coordinators should institutionalize mentorship responsibilities as part of national workforce development strategies rather than treating them as voluntary or add-on roles.

The study also revealed gaps in organizational and resource support. Many mentors reported inadequate financial incentives and limited access to digital or educational tools needed for effective mentorship. Prior research indicates that mentorship programs with structured support systems and adequate funding achieve stronger and more sustainable outcomes (20). FETP programs should therefore establish formal mentorship frameworks that include small stipends, logistical assistance, and standardized materials to guide mentor–mentee interactions. Strengthening institutional backing not only improves mentor performance but also signals organizational recognition of mentorship as a valued professional function.

Communication and feedback emerged as additional areas requiring improvement. Mentors reported difficulties in maintaining consistent communication with mentees, partly due to inadequate digital communication infrastructure and, at times, low responsiveness from trainees. Effective communication is essential for building trust, providing timely guidance, and fostering accountability. Establishing clear communication protocols—such as scheduled check-ins, structured reporting templates, and the use of digital communication platforms—can help standardize interactions and maintain engagement, especially in geographically dispersed programs.

Equally important is the finding that formal feedback mechanisms were often absent. Without structured feedback, mentors have limited opportunity to assess mentees' progress or receive constructive input about their own performance. The literature highlights that well-designed feedback systems enhance learning outcomes, mentor satisfaction, and program effectiveness (21). FETP programs should institute formal, two-way feedback processes—using standardized evaluation forms and periodic debriefs—to ensure that mentorship remains a dynamic, reciprocal, and evidence-informed process.

Training and capacity-building needs identified in this study further reinforce the importance of continuous mentor development. Although most mentors had received mentorship training at some point, fewer than half had done so recently, indicating the need for ongoing refresher courses. Training priorities such as mentoring skills, communication, leadership, and feedback techniques reflect the complex interpersonal and managerial roles mentors must play beyond technical supervision. Tailored training modules, delivered through interactive workshops and case-based learning, can enhance mentors' ability to provide individualized guidance, manage conflicts, and support mentees' professional growth. This approach aligns with global evidence showing that participatory and practice-oriented mentor development leads to improved outcomes for both mentors and mentees (22). Establishing regional mentor training hubs, potentially coordinated through EMPHNET, could help standardize competencies, promote quality assurance, and foster cross-country learning.

The findings also underscore the need for FETP programs to adopt a systems perspective toward mentorship. Mentorship should not depend solely on individual goodwill but be embedded within institutional frameworks that define clear expectations, provide tangible support, and recognize contributions. Creating mentorship guidelines and competency frameworks will help standardize roles and ensure consistency across programs. Recognition mechanisms—such as professional certification, awards, or advancement credits—can incentivize long-term mentor engagement and attract new mentors to the system. Integrating mentorship indicators into program monitoring and evaluation would allow FETP leadership to systematically assess mentor performance, identify gaps, and adjust training strategies accordingly.

This study has limitations. The reliance on self-reported data may introduce recall and social desirability biases. Selection bias may also have occurred if mentors who were more engaged or satisfied were more likely to respond. Moreover, the absence of qualitative data limited the exploration of contextual nuances that could enrich interpretation.

In conclusion, this study identified key strengths and gaps in FETP mentorship. Strengthening mentorship within FETPs requires a comprehensive approach that combines institutional commitment, structured mentor training, adequate resources, and continuous feedback and recognition. By addressing the barriers identified and leveraging the strong motivation and expertise of mentors, FETP programs can ensure sustainable, high-quality mentorship that contributes to building a competent and resilient public health workforce in the EMR. Providing periodic refresher training, particularly for mentors with limited experience, can help improve mentorship quality. Establishing formal mentorship guidelines that clearly define roles, responsibilities, and expectations will ensure a more structured approach. Allocating dedicated time for mentorship activities is necessary to reduce conflicts with other professional responsibilities. Strengthening financial and logistical support, including access to essential technological tools, can enhance the overall mentorship experience. Encouraging active participation in CPD programs will keep mentors updated on epidemiology and public health advancements. Recognizing and rewarding mentors for their contributions can serve as motivation for sustained participation. Implementing structured feedback systems will allow for continuous assessment of mentorship effectiveness and identification of areas for improvement, and promoting regular two-way feedback between mentors and mentees can further enhance engagement and support professional growth. Future research should include qualitative interviews and extend the analysis to mentees to capture both sides of the mentorship experience and identify alignment or gaps between mentor and mentee perspectives.

Addressing time constraints, resource limitations, and training needs is essential to enhancing mentorship effectiveness. Future efforts should prioritize structured mentor development, clear guidelines, and robust support systems to sustain impactful mentorship within FETPs.

## Data Availability

The raw data supporting the conclusions of this article will be made available by the authors, without undue reservation.
